# 684. Impact of peer-comparison feedback on hospitalists’ antibiotic prescribing of broad-spectrum antibiotics: a stepped wedge randomized clinical trial

**DOI:** 10.1093/ofid/ofaf695.223

**Published:** 2026-01-11

**Authors:** Scott Fridkin, Lucy S Witt, Zanthia Wiley, Hasan Shabbir, Radhika Asrani, C Christina Mehta, K Ashley Jones, Julianne Gent, Raymund Dantes, Chad Robichaux, Jessica Howard-Anderson, Jesse T Jacob

**Affiliations:** Georgia Emerging Infections Program, Decatur, GA; Emory University School of Medicine, Atlanta, GA, Atlanta, Georgia; Emory University, Atlanta, Georgia; Emory University School of Medicine, Atlanta, GA; Emory Universtiy, Atlanta, Georgia; Emory University, Atlanta, Georgia; Atlanta Women’s Interagency HIV; Emory University, Rollins School of Public Health, Department of Biostatistics and Bioinformatics, Atlanta, GA; Emory Healthcare, Atlanta, Georgia; Emory Healthcare, Atlanta, Georgia; Emory Healthcare, Atlanta, Georgia; Emory University, Atlanta, Georgia; Emory University, Atlanta, Georgia; Emory University School of Medicine, Atlanta, GA

## Abstract

**Background:**

Antibiotic prescribing report feedback is relatively untested in inpatient settings; we assessed the efficacy of a pilot program benchmarking inpatient antibiotic prescribing among hospitalists in a large academic healthcare network.

**Figure:**
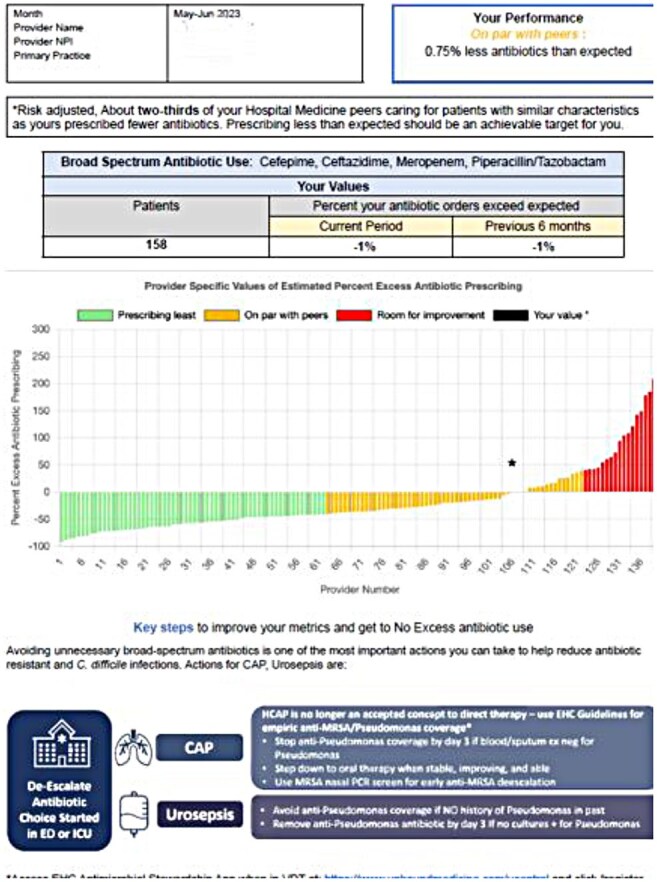
Sample Peer-Comparison Antibiotic Prescribing Report

**Figure:**
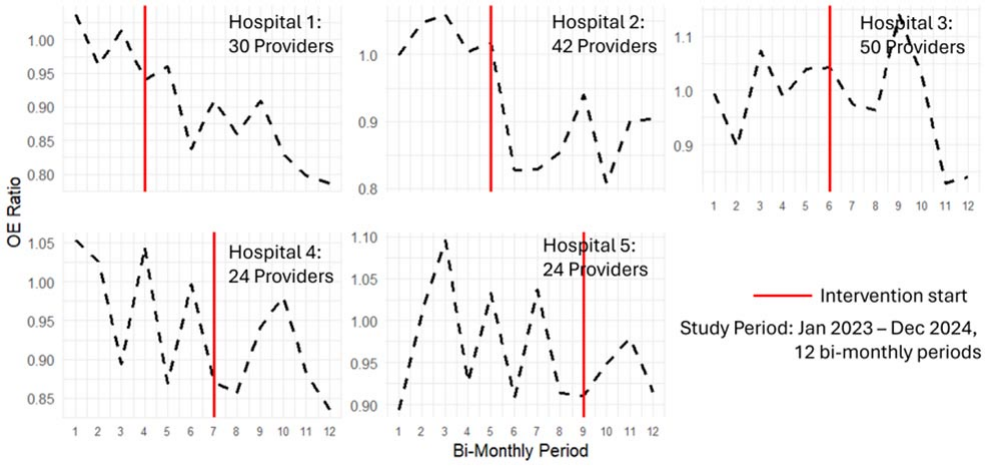
Mean Observed to Expected Ratio of Days of Therapy, Among all Providers, for Each Facility, Over Time Each period is 2-months, the vertical red line is the time of the intervention (providing reports via email).

**Methods:**

Linked 2023-2024 billing and prescribing data from hospitalists at 5 hospitals were pooled into 12 bi-monthly periods. Expected days of therapy (DOT) for NHSN–broad spectrum-hospital onset (BS-HO) agents were estimated using previously published linear mixed models adjusting for patient characteristics; observed to expected ratios (OERs) were calculated for each provider-period. Peer-comparative reports (Figure 1) were disseminated via email every two months in a stepped wedge cluster trial design starting at month 6 in a random order. An emailed survey was sent to each provider after the third report as an implementation check. Negative binomial mixed effects regression modeling was used to assess the effect of the intervention on providers’ prescribing rate, accounting for time-period, repeated measures by providers, clustering of providers within hospitals and patient characteristics.

**Results:**

We included 165 hospitalists (range 24-50 per hospital) for a total of 1687 provider-periods; sequentially starting the intervention after 3 periods at hospital 1. Per period, providers cared for a mean 132 patients (range 63-306) over 330 patient-days (range 165-478). The median OER overall was 0.94 (0.98 pre-intervention to 0.88 post intervention); OERs trended downward (Figure 2). In the model fully adjusting for patient-days with sepsis (incidence rate ratio [IRR] 1.04; 95% CI 1.0-1.08), ESRD (IRR 1.09; 95% CI 1.05-1.14), UTI (IRR 1.0; 95% CI 0.97-1.05), and time-period (IRR 0.99, 95% CI 0.98-1.0), the intervention was not significantly associated with lower prescribing rates (IRR 0.97; 95% CI 0.91-1.04). These findings were unchanged when limiting the analytic cohort to only the 76 providers confirmed to have understood the reports.

**Conclusion:**

In this cluster trial of a quality improvement effort, the emailed prescribing reports did not significantly change hospitalist’s prescribing rates of BS-HO antibiotics. Evaluating reasons for lack of efficacy and additional efforts to augment utility of the reports is warranted.

**Disclosures:**

All Authors: No reported disclosures

